# Coronavirus Receptor Expression Profiles in Human Mast Cells, Basophils, and Eosinophils

**DOI:** 10.3390/cells13020173

**Published:** 2024-01-17

**Authors:** Lina Degenfeld-Schonburg, Irina Sadovnik, Dubravka Smiljkovic, Barbara Peter, Gabriele Stefanzl, Clemens Gstoettner, Peter Jaksch, Konrad Hoetzenecker, Clemens Aigner, Christine Radtke, Michel Arock, Wolfgang R. Sperr, Peter Valent

**Affiliations:** 1 Department of Internal Medicine I, Division of Hematology & Hemostaseology, Medical University of Vienna, 1090 Vienna, Austria; lina.degenfeld-schonburg@meduniwien.ac.at (L.D.-S.);; 2 Ludwig Boltzmann Institute for Hematology and Oncology, Medical University of Vienna, 1090 Vienna, Austria; 3 Department of Plastic, Reconstructive and Aesthetic Surgery, Medical University of Vienna, 1090 Vienna, Austria; 4 Department of Thoracic Surgery, Medical University of Vienna, 1090 Vienna, Austriaclemens.aigner@meduniwien.ac.at (C.A.); 5 Laboratory of Hematology, Pitié-Salpêtrière Hospital, 75651 Paris, France; michel.arock@aphp.fr

**Keywords:** mast cells, basophils, eosinophils, SARS-CoV-2 receptors, inflammation

## Abstract

A major problem in SARS-CoV-2-infected patients is the massive tissue inflammation in certain target organs, including the lungs. Mast cells (MC), basophils (BA), and eosinophils (EO) are key effector cells in inflammatory processes. These cells have recently been implicated in the pathogenesis of SARS-CoV-2 infections. We explored coronavirus receptor (CoV-R) expression profiles in primary human MC, BA, and EO, and in related cell lines (HMC-1, ROSA, MCPV-1, KU812, and EOL-1). As determined using flow cytometry, primary MC, BA, and EO, and their corresponding cell lines, displayed the CoV-R CD13 and CD147. Primary skin MC and BA, as well as EOL-1 cells, also displayed CD26, whereas primary EO and the MC and BA cell lines failed to express CD26. As assessed using qPCR, most cell lines expressed transcripts for CD13, CD147, and ABL2, whereas ACE2 mRNA was not detectable, and CD26 mRNA was only identified in EOL-1 cells. We also screened for drug effects on CoV-R expression. However, dexamethasone, vitamin D, and hydroxychloroquine did not exert substantial effects on the expression of CD13, CD26, or CD147 in the cells. Together, MC, BA, and EO express distinct CoV-R profiles. Whether these receptors mediate virus–cell interactions and thereby virus-induced inflammation remains unknown at present.

## 1. Introduction

The SARS-CoV-2 pandemic has distorted our healthcare systems. In fact, due to a lack of knowledge about disease pathogenesis and the difficulties in implementing optimal management plans in various countries during the initial phase of the pandemic, COVID-19-induced morbidity and mortality were rather high [1,2,3]. One major issue is the massive tissue inflammation that is often seen in SARS-CoV-2-infected individuals. Recent data suggest that during coronavirus (CoV) infections, the activation of pro-inflammatory effector cells in certain target organs, such as the lung, may play a crucial role and may determine the severity of the resulting pathology [3,4,5,6,7,8,9]. For example, in severe COVID-19 pneumonia, massive local tissue inflammation and consecutive organ damage are considered to contribute essentially to morbidity and mortality in affected patients. Therefore, several attempts have been made to block inflammation in these patients [10,11,12,13,14]. However, despite numerous studies, it remains uncertain what cells and mediators play a major role in organ damage and must be blocked to sufficiently interrupt tissue inflammation in COVID-19 pneumonia and other corona-induced pathologies. However, despite numerous studies, it remains uncertain what cells and mediators play a major role in organ damage and must be blocked to sufficiently interrupt tissue inflammation in COVID-19 pneumonia and other corona-induced pathologies. A related question is which type of cells express which type of coronavirus receptors (CoV-R) and how the expression and function of these receptors are regulated.

Mast cells (MC), basophils (BA), and eosinophils (EO) are key effector cells in inflammatory reactions [15,16,17,18,19,20,21,22]. These cells express activation-linked cell surface antigens and can produce several pro-inflammatory mediators and cytokines [15,16,17,18,21]. During hypersensitivity reactions and acute or chronic inflammation, MC, BA, and EO may release their pro-inflammatory substances into local tissue sites, thereby contributing to tissue inflammation and tissue damage [15,16,18,19,20].

A number of previous and more recent studies have shown that MC, BA, and EO play an active role in several infectious diseases, including bacterial and viral infections [16,19,20,23,24,25,26,27,28]. Especially in viral disorders, these cells may play an active role as effector cells and sometimes even as virus reservoirs [23,24,26,27,28]. It is also worth noting that viral infections, including SARS-CoV-2 infections, often manifest in organ systems (lung, skin, and gastrointestinal tract) that are rich in MC and are often infiltrated by EO during inflammatory reactions and active infections. However, only little is known about the role of MC, BA, or EO in SARS-CoV-2-induced inflammation. In addition, little is known about the distribution of CoV-R on MC, BA, and EO.

Several CoV-R have been identified in recent decades, including angiotensin-converting enzyme 2 (ACE2), CD13, CD26, and CD147 [29,30,31,32,33,34]. A summary of known CoV-R is shown in Table 1. These receptors are expressed in various target cells, such as epithelial cells, endothelial cells, or macrophages [30,31,32,33]. However, so far, little is known about the expression of these CoV-R on pro-inflammatory effector cells in health and disease. The aims of the present study were to investigate the expression and regulation of CoV-R in human MC, BA, and EO.

## 2. Materials and Methods

### 2.1. Reagents

Vitamin D was purchased from Selleck Chemicals (Houston, TX, USA) and dexamethasone and hydroxychloroquine from Sigma-Aldrich (St. Louis, MO, USA). Stock solutions of drugs were prepared by dissolving in dimethylsulfoxide (DMSO) (Merck, Darmstadt, Germany). RPMI 1640 medium, Iscove’s Modified Dulbecco’s Medium (IMDM), and antibiotics (penicillin + streptomycin) were purchased from Lonza (Verviers, Belgium), fetal calf serum (FCS) from Gibco, Life Technologies (Paisley, UK), alpha-thioglycerol from Sigma-Aldrich, and amphotericin B from PAN Biotech (Aidenbach, Germany). Recombinant human stem cell factor (SCF) was obtained from Peprotech (Cranbury, NJ, USA). Collagenase type II was purchased from Merk (Darmstadt, Germany) or Stemcell Technologies (Vancouver, BC, Canada). A specification of monoclonal antibodies (mAb) used in this study is shown in Supplementary Table S1. Fc-blocking reagent was obtained from Militenyi Biotec (Bergisch Gladbach, Germany).

### 2.2. Isolation of Primary MC, BA, and EO

Primary peripheral blood (PB) was obtained from healthy donors (n = 13) and patients with hypereosinophilia (HE) (n = 3), including one with reactive HE and chronic kidney disease, one with a lymphocyte-variant of the hypereosinophilic syndrome (HES), and one with a myelodysplastic/myeloproliferative neoplasm with HE (MPN/MDS-Eo). In addition, the PB of 2 patients suffering from a SARS-CoV-2 infection was examined. Bone marrow (BM) was collected from 3 patients with systemic mastocytosis (SM), one with cutaneous mastocytosis (CM), one with hereditary alpha-tryptasemia (HαT), one with unexplained pancytopenia, one with chronic spontaneous urticaria, one with idiopathic HE and chronic kidney disease, and one with Hodgkin lymphoma without BM involvement. All PB and BM samples were obtained during diagnostic routine investigations. The patients’ characteristics are shown in Table 2. Freshly obtained samples were analyzed using flow cytometry or subjected to isolation of mononuclear cells (MNC) using Ficoll. Lung MC were obtained from patients undergoing lung transplantation and skin MC from patients undergoing abdominoplasty at the University Hospital of Vienna (Medical University of Vienna). Tissue MC were isolated essentially as described [35,36,37]. In brief, tissue was cut into small fragments, washed extensively, and left overnight at 4 °C in Ca/Mg-free Tyrode’s buffer. Then, tissue fragments were washed and incubated in 1.5 mg/mL collagenase type II at 37 °C for 2 × 90 min. Digestion was stopped by adding FCS. Isolated cells were filtered through a 70 μm cell strainer (Corning Incorporated, Corning, NY, USA) to obtain single cell suspensions. Isolated cells were washed and cultured in RPMI 1640 medium supplemented with 10% FCS, 1% penicillin+steptomycin, 1% amphotericin B, and 25 ng/mL SCF at 37 °C and 5% CO_2_. All donors provided their written informed consent before PB or tissue samples (BM, skin, lung) were collected. The study was approved by the ethics committee of the Medical University of Vienna (1184/2014 and 1040/2022).

### 2.3. Cell Lines

The FIP1L1::PDGFRA+ eosinophilic cell line EOL-1 was purchased from the German Collection of Microorganisms and Cell Cultures (DSMZ, Braunschweig, Germany). EOL-1 cells were maintained in RPMI 1640 medium supplemented with 20% heat-inactivated FCS and antibiotics at 37 °C and 5% CO_2_. The multipotent human BCR::ABL1+ BA cell line KU812 was kindly provided by Dr. K. Kishi (Niigata University, Niigata, Japan) [38]. The human MC leukemia cell line HMC-1 was kindly provided by Dr. J. H. Butterfield (Mayo Clinic, Rochester, MN, USA) [39]. Two sub-clones were used, namely, HMC-1.1 exhibiting KIT V560G, and HMC-1.2 harboring KIT V560G and KIT D816V [40,41]. HMC-1 cells were grown in IMDM with 10% FCS, alpha-thioglycerol, and antibiotics. The recently established human MC lines ROSA^KIT WT^, ROSA^KIT D816V^, and ROSA^KIT K509I^ [42], and four MCPV-1 subclones (MCPV-1.1, MCPV-1.2, MCPV-1.3, and MCPV-1.4) were cultured in IMDM containing 10% FCS. MCPV-1 cells are derived from MC-committed cord blood progenitors transformed by oncogenic *H-TERT*, *large-T*, and *HRAS* G12V [43]. ROSA^KIT D816V^ and ROSA^KIT K509I^ cells were established by lentiviral transduction as reported in [42]. To maintain ROSA^KIT WT^, MCPV-1.1, MCPV-1.2, MCPV-1.3, and MCPV-1.4 cells, SCF-containing supernatants (10%) of Chinese hamster ovary cells transfected with the murine *scf* (kl) gene (CHOKL) were used as reported in [42,43].

### 2.4. Evaluation of Surface Expression of CoV-R on Cell Lines and Primary Leukocytes Using Flow Cytometry

To analyze the expression of CoV-R on eosinophilic (EOL-1), basophilic (KU812), and MC-related (HMC-1.1, HMC-1.2, ROSA^KIT WT^, ROSA^KIT D816V^, ROSA^KIT K509I^, MCPV-1.1−1.4) cell lines, surface staining was performed with flow cytometry using fluorochrome-conjugated mAb as described [43]. In brief, cells were first pre-incubated with Fc-block to limit unspecific binding. Then, cells were incubated with PE-conjugated CD-clustered mAb against CD13 (aminopeptidase-N, ANPEP), CD26 (dipeptidylpeptidase IV, DPPIV), and CD147 (basigin, BSG) at room temperature for 15 min, washed in PBS, and analyzed on a Cytoflex S (Beckman Coulter, Brea, CA, USA).

To analyze the expression of CoV-R on primary MC, skin-, lung- and BM cells were incubated with PE-conjugated mAb against CD13, CD26, and CD147. In BM samples, MC were defined as CD117+/CD45+/CD34− cells, and in lung and skin samples, MC were defined as CD117+/CD45+ cells. Accordingly, BM cells were also incubated with PE-Cy7-labeled mAb against CD117 (KIT), APC-Cy7- or V500-conjugated mAb against CD45, and a PB-conjugated mAb against CD34, and lung and skin samples were incubated with mAb against KIT and CD45. In the case of BM samples, erythrocyte lysis was performed by adding lysis buffer (Becton Dickinson; 1:10 diluted in dH2O) for 15 min. Stained cells were examined using multicolor flow cytometry as reported in [35,43,44]. To assess the expression of CoV-R on PB EO (Siglec-8+ cells) and PB BA (CD203c+/CD123+/CD45+/CD14− cells), whole blood samples were examined as reported [44,45]. In brief, cells were incubated with PE-conjugated mAb against Siglec-8, CD13, CD26, or CD147 as well as FITC-conjugated mAb against CD14, APC-Cy7-conjugated mAb against CD45, APC-conjugated mAb against CD203c, and PE-Cy7-conjugated mAb against CD123. Cells were incubated in the dark at room temperature for 15 min. Then, cells were washed and analyzed using multicolor flow cytometry. All flow cytometry staining experiments were performed on a Cytoflex S (Beckman Coulter) and analyzed using FlowJo software, version 10.7.1 (TreeStar, Ashland, OR, USA). The mAb used in this study are shown in Supplementary Table S1.

### 2.5. Effects of Various Drugs on Expression of CoV-R on MC, BA, and EO

To evaluate the potential drug effects on CoV-R expression on MC-related cell lines (HMC-1.1, HMC-1.2, ROSA^KIT WT^, ROSA^KIT D816V^, ROSA^KIT K509I^, and MCPV-1.1-1.4), the basophilic cell line KU812, and the eosinophilic cell line EOL-1, these cells were incubated in a control medium or a medium supplemented with 1 µM dexamethasone, 10 µM hydroxychloroquine, or 5 µM vitamin D at 37 °C for 24 h. In EOL-1 cells, hydroxychloroquine was found to downregulate the expression of CD26 in our screening experiment. Therefore, we applied different concentrations of this drug (1–50 µM) on EOL-1 cells in subsequent experiments. After drug incubation, surface staining was performed using PE-conjugated mAb against CD13, CD26, and CD147, and then, flow cytometry was performed following the published techniques [43].

### 2.6. Quantitative PCR (qPCR)

RNA was isolated from MC-related cell lines (HMC-1.1, HMC-1.2, ROSA^KIT WT^, ROSA^KIT D816V^, ROSA^KIT K509I^, and MCPV-1.1 through 1.4), the BA cell line KU812, and the EO-related cell line EOL-1. cDNA was synthesized using Moloney murine leukemia virus reverse transcriptase and random primers (Invitrogen, Carlsbad, CA, USA). To confirm the expression of CD13 (ANPEP), CD26 (DPPIV), CD147 (BSG), ACE2, ABL1, and ABL2 mRNA in our cell lines, qPCR experiments were conducted as reported in [44,46,47] using primers listed in Supplementary Table S2. qPCR was performed using iTaq Universal SYBR Green Supermix and plasmid standards. CD13, CD26, CD147, ACE2, ABL1, and ABL2 mRNA copy numbers were normalized to beta-glucuronidase (GUSB) mRNA copy numbers and expressed as a percent of GUSB. Technical details are described in the supplement.

### 2.7. Statistical Analysis

To determine the level of significance in differences of the expression of CoV-R between drug-exposed cells and control cells, one-way ANOVA followed by Bonferroni’s post hoc test for multiple comparisons was applied. To test the differences in the expression of CoV-R between two groups, the paired Student’s *t*-test was used. Results were considered to be significantly different when *p* < 0.05.

## 3. Results

### 3.1. MC, BA, and EO Display Distinct Profiles of Cell Surface CoV-R

**Figure 1:**
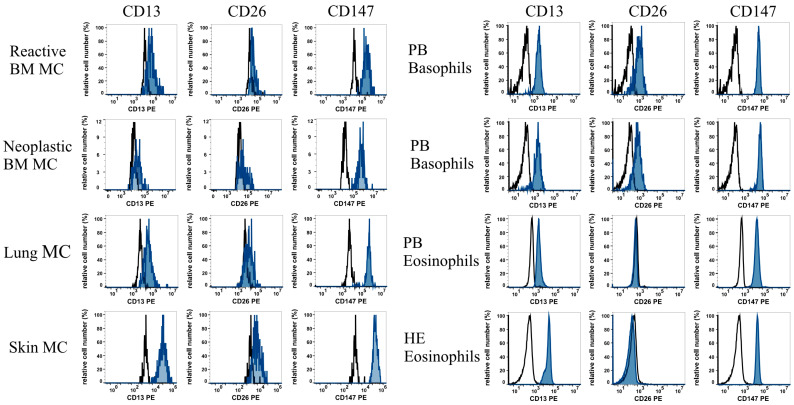
Expression of coronavirus receptors on primary human mast cells, basophils, and eosinophils. Reactive (non-neoplastic) BM MC (CD117+/CD45+/CD34−), neoplastic BM MC (CD117+/CD45+/CD34−), lung and skin MC (CD117+/CD45+), healthy PB basophils (CD203+/CD123+/CD45+/CD14−), healthy PB eosinophils (Siglec-8+), and PB eosinophils obtained from patients diagnosed with eosinophilia (Siglec-8+) were stained with PE–conjugated antibodies against three CoV-R, CD13 (aminopeptidase N; clone WM15), CD26 (dipeptidylpeptidase IV; clone M-A261), and CD147 (basigin; clone HIM-6). Antibody reactivity was analyzed using multicolor flow cytometry and is shown in blue histograms. The black open histograms show the reactivity with the isotype–matched control antibody. Abbreviations: BM, bone marrow; CD, cluster of differentiation; CoV-R, coronavirus receptor; HE, hypereosinophilia; MC, mast cells; PB, peripheral blood; and PE, phycoerythrin.

As assessed with flow cytometry, primary lung and skin MC, BM MC, PB BA, and PB EO expressed the CoV-R CD13 and CD147 (Table 3, Figure 1). Primary BM MC, skin MC, and BA also displayed CD26, whereas primary EO did not express CD26 (Table 3, Figure 1). PB BA expressed higher levels of CD26 compared to those of tissue MC (Figure 1). The highest expression levels of CD13 were detected on skin MC, followed by lung and BM MC. Although EO obtained from patients with HE exhibited slightly higher levels of CD13 compared to EO in normal donors, no significant differences were found in the expression of CD13 and CD147 when comparing healthy controls (n = 13) with HE patients (n = 3) (*p* > 0.05 as determined by Student’s *t*-test). Finally, we examined CoV-R expression on PB BA and PB EO in two patients with an ongoing SARS-CoV-2 infection. However, we did not find differences in CoV-R expression levels when comparing BA and EO of healthy controls with BA and EO obtained from SARS-CoV-2-infected patients.

### 3.2. Expression of CoV-R in MC, BA, and EO Cell Lines

**Figure 2:**
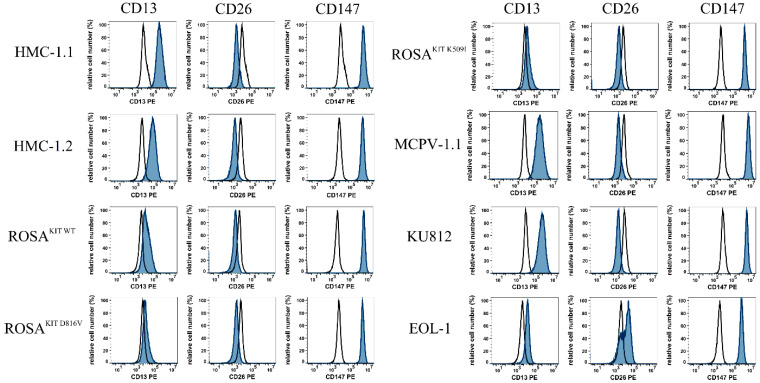
Expression of coronavirus receptors on mast cell-, basophil-, and eosinophil-related cell lines. The MC-related cell lines HMC-1.1, HMC-1.2, ROSA^KIT WT^, ROSA^KIT D816V^, ROSA^KIT K509I^, and MCPV-1.1, the eosinophilic cell line EOL-1, and the basophilic cell line KU812 were stained with PE-conjugated antibodies against three CoV-R, CD13, CD26, and CD147. Antibody reactivity was analyzed using flow cytometry and is shown in blue histograms, and the isotype–matched control antibody is shown in black open histograms. Abbreviations: CD, cluster of differentiation; CoV-R, coronavirus receptor; and PE, phycoerythrin.

As assessed using flow cytometry, all MC lines tested as well as the BA cell line KU812 and the EO cell line EOL-1 stained positive for CD13 and CD147 (Table 3, Figure 2). EOL-1 cells also expressed CD26, whereas the MC lines tested and KU812 cells stained negative for CD26 (Table 3, Figure 2). CoV-R expression in cell lines was confirmed with qPCR. Again, almost all cell lines expressed mRNA specific for CD13 and CD147, whereas most of these cells, except EOL-1, failed to express CD26 mRNA. We were also able to show that these cells express measurable levels of ABL1 and ABL2 mRNA. In contrast, ACE2 mRNA was not expressed in these cells. A summary of qPCR data obtained with our MC, BA, and EO cell lines is shown in Table 4.

### 3.3. Effects of Pharmacologic Inhibitors on Expression of CoV-R on MC, BA, and EO

In an attempt to identify agents that suppress the expression of CoV-R on MC, BA, or EO, we examined the effects of dexamethasone, vitamin D, and hydroxychloroquine on the expression of CoV-R on HMC-1, ROSA, MCPV-1, KU812, and EOL-1 cells. However, at the tested concentrations, these drugs showed no substantial effects on the surface expression of CoV-R expression in these cells (Table 5). In fact, we observed a slight downregulation of CD13 and CD147 on HMC-1.1 cells after incubation with dexamethasone (Table 5). In addition, we found a slight downregulation of CD26 on EOL-1 cells after treatment with hydroxychloroquine (Table 5; Supplementary Figure S1). However, over the dose range that was tested, hydroxychloroquine did not reach an IC_50_ value (Supplementary Figure S1).

## 4. Discussion

MC, BA, and EO are major pro-inflammatory effector cells of the immune system [15,16,17,18,19,20,21,22]. These cells play a key role in various infectious diseases, including viral infections [23,24,26,27,28]. COVID-19 is characterized by SARS-CoV-2-induced organ damage in certain target organs, such as the lung or the gastrointestinal tract [3,4,5,6,7,8,9]. These organs are also a rich source of MC. In addition, during an inflammatory reaction or infection, EO and BA may invade and increase in number in these organs. Recent data suggest that MC, BA, and EO are involved in SARS-CoV-2 infections. Indeed, in patients with SARS-CoV-2 infection, the accumulation and activation of MC and EO have been described [48,49,50,51,52]. It has also been described that these cells may be involved in CoV-induced organ damage. For example, increased levels of eosinophil mediators during SARS-CoV-2 infection have been reported, indicating that eosinophil activation occurs in these patients [49]. It has also been described that the targeting of MC and EO with an anti-Siglec-8 antibody leads to an improvement of organ damage and inflammation during SARS-CoV-2 infection [49]. However, so far, the mechanisms underlying COVID-19-mediated tissue damage and how MC, BA, and EO may contribute to these pathologies remain largely unknown. We have established the CoV-R expression profiles for human MC, BA, and EO. Our data show that MC, BA, and EO, as well as the respective cell lines, express the CoV-R CD13 and CD147 on their surface. In addition, these cells express ABL1 and ABL2 but do not express ACE2 mRNA. Finally, some of the inflammatory effector cells, including blood BA and tissue MC, expressed the CoV-R CD26.

CD13, also known as aminopeptidase N (ANPEP), is a cell surface enzyme that was described as a receptor for the human CoV 229E [32,53]. This receptor is expressed in tissue cells in various organs, including the gastrointestinal tract, kidney, and lung epithelium, and also in various hematopoietic cells, such as immature myeloid cells, monocytes, and granulocytes [54,55,56]. More recently, CD13 has also been considered as a potential target of small-molecule type targeted drugs and immunotherapy in myeloid neoplasms [56,57,58,59]. In our study, we show that primary human MC, BA, and EO as well as the tested leukemic cell lines display cell surface CD13. These results are in line with earlier studies [60,61,62,63,64]. We were also able to confirm the expression of CD13 at the mRNA level in our MC and BA cell lines using qPCR. However, unexpectedly, we were not able to confirm the expression of CD13 mRNA in the eosinophilic cell line EOL-1, whereas these cells clearly reacted with antibodies against CD13 in our flow cytometry analyses. One explanation for this discrepancy may be a very low production rate of CD13 in EOL-1 cells as well as the stability of surface expression of this antigen on these cells. Alternatively, the CD13 mAb produced a non-specific staining reaction on EOL-1 cells. However, this possibility seems unlikely as the mAb used (WM15) was tested in Human Leukocyte Differentiation Antigen Workshops and produced positive and negative staining reactions in all tested control cells.

Dipeptidylpeptidase IV (DPPIV, CD26) is expressed on CML stem cells, in CML- and normal BA, and in skin MC [35,47,65]. It has also been described that CD26 serves as a receptor for the Middle East respiratory syndrome coronavirus (MERS-CoV) [33]. We were able to show that CD26 is expressed on tissue MC and PB BA but not on PB EO. In contrast to our findings on primary cells, the eosinophilic cell line EOL-1 stained positive for CD26 and expressed CD26 mRNA. In contrast, the MC lines as well as the BA cell line KU812 stained negative for CD26. The reason for the differential expression of CD26 on EOL-1 cells (positive) and PB EO (negative) remains unknown. One explanation for this may be that CD26 is only expressed on immature eosinophil precursor cells and in immature cell lines, such as EOL-1, but not on mature EO. Alternatively, the EOL-1 cell lines expressed CD26 in an aberrant or in an oncogene-dependent (FIP1L1::PDGFRA-induced) manner. Unfortunately, we were not able to test this hypothesis in the current study, as we could not examine EO derived from patients with FIP1L1::PDGFRA-mutated myeloid neoplasms. Finally, the expression of CD26 on EO may be an activation-related event. However, again, we were not able to test cells derived from patients with HE syndromes (HES) where EO are often in an activated state.

Another known virus receptor is CD147, also termed as basigin. This transmembrane glycoprotein is a receptor for different viruses, including measles virus and human immunodeficient virus (HIV) [66,67]. Recently, CD147 was also identified as a receptor for SARS-CoV-2 [34,68]. In the current study, we found that MC, BA, and EO, as well as the related cell lines, express high amounts of cell surface CD147. In addition, we were able to show that these cells display a substantial amount of CD147 mRNA. These results confirmed previous data showing the expression of CD147 in human MC and BA [69,70].

The major SARS-CoV-2 receptor appears to be ACE2 [68,71,72]. This receptor is expressed on various target cells, including alveolar and other epithelial cells. Inflammatory effector cells may also express ACE2 on their surface under certain conditions. However, in our experiments, all MC, BA, and EO cell lines examined were found to lack ACE2 mRNA. This may be due to the fact that ACE2 is only produced in activated effector cells or only after exposure to viral antigen. Indeed, it has been described that under certain conditions, MC display ACE2. For example, the human MC line HMC-1 reportedly expresses measurable levels of ACE2 after activation by phorbol 12-myristate 13-acetate (PMACI) [73]. Whether activated human tissue MC can display ACE2 remains unknown. So far, resting MC were found to lack ACE2 mRNA [74,75], which confirms the data obtained in this study.

Apart from cell surface receptors mediating viral entry into target cells, some intracellular receptor sites binding CoV have been described. For example, ABL2 has been reported to be an important cofactor for the viral uptake, and the ABL2 inhibitor imatinib was found to prevent the coronavirus fusion with the endosomal membrane [76]. Therefore, we also tested the expression of ABL1 mRNA and ABL2 mRNA in our study. Indeed, we found that all MC lines, the BA line KU812, and the EO cell line EOL-1 express transcripts for ABL1 and ABL2.

Although CD13, CD26, CD147, and ABL2 have been described to bind to certain CoV-R, it remains unknown whether coronaviruses can indeed interact with and can transfect MC, BA, and/or EO through these antigens. In particular, it would be of interest to learn whether these cells or the respective cell lines can be infected with SARS-CoV-2 or other coronaviruses through the surface receptors that we have identified, such as hCov-229E via CD13 or MERS-CoV through CD26. These experiments are planned and will be the subject of a forthcoming study.

A number of anti-inflammatory and/or anti-viral drugs have been tested for their clinical efficacy in patients with COVID-19. Some of these agents may also act on MC, BA, and/or EO. We attempted to decipher whether dexamethasone, vitamin D, and hydroxychloroquine can downregulate the expression of CoV-R on MC, BA, and EO. However, although the minor inhibitory effects of hydroxychloroquine on CD26 expression in EOL-1 cells were seen, overall, no substantial suppression of CoV-R expression on MC, BA, or EO could be demonstrated with the applied drugs in this study. Such complete suppression could interfere with virus entry but may only be reached when applying very high doses of these drugs or even drug combinations. However, such therapy may also cause side effects and could even exert toxic rather than immunosuppressive effects on inflammatory effector cells. Therefore, we did not apply such high concentrations in our experiments. Another strategy would be to test other classes of anti-inflammatory drugs or even (antibody-based) drugs that can directly block these CoV-R on MC, BA, and EO. Some of these inhibitors could be gliptins (DDPIV/CD26-targeting agents), the aminopeptidase (CD13) inhibitor bestatin, or the humanized anti-CD147 antibody meplazumab. A most attractive approach may be to combine such inhibitors. However, further studies are needed to show that such drug combinations are tolerable in patients and can completely block viral uptake and viral spread in MC, BA, and EO. It would also be of interest to study the functional role of the receptors identified on MC, BA and EO. This would require more studies including the exposure of cells to recombinant CoV antigens, such as SARS-CoV-2 spike protein, and the subsequent testing of cell activation and expression of surface antigens. These experiments are the subject of a forthcoming project.

In conclusion, our study provides novel insights into the distribution of various CoV-R in MC, BA, and EO, which may contribute to our understanding of the potential role of these inflammatory effector cells in CoV infections. In addition, these data may support the development of drugs interfering with CoV-R expression and, thus, the viral infection of major inflammatory effector cells.

## Figures and Tables

**Table 1:** Receptors for human coronaviruses (CoV) and related antigens.

Antigen	CoV
ACE2	SARS-CoV, SARS-CoV-2, hCoV-NL63
ABL2	SARS-CoV, MERS-CoV
CD13 = Aminopeptidase N	hCoV-229E
CD26 = Dipeptidylpeptidase IV	MERS-CoV
CD147 = Basigin	SARS-CoV-2
9-O-Acetylated Sialic Acid	hCoV-OC43, hCoV-HKU1

Abbreviations: ACE2, angiotensin-converting enzyme 2; (h)CoV, (human) coronavirus; MERS-CoV, Middle East respiratory syndrome coronavirus; and SARS-CoV, severe acute respiratory syndrome coronavirus.

**Table 2:** Patients’ characteristics.

Patient Number	Age (Years)	Sex (m/f)	Diagnosis	Sample PB or BM	Serum Tryptase (ng/mL)	Hb (g/dL)	WBC (G/L)	PLT (G/L)	% EO in PB	% MC in MNC ^a^	% MC in BM Smears ^b^	% MC in BM Histology
#1	61	f	Pancytopenia *	BM	n.a.	7.9	2.43	27	0	0.02%	<1	n.a.
#2	32	f	Chronic Kidney Disease and Idiopathic Hypereosinophilia	BM	26.8	10.2	12.72	359	32	0.07%	<1	n.a.
#3	45	m	Chronic spontaneous Urticaria	BM	5	13.1	7.74	230	9	0.006%	<1	n.a.
#4	52	m	Hodgkin Lymphoma	BM	n.a.	12.3	10.05	330	4	0.003%	<1	n.a.
#5	24	f	Cutaneous Mastocytosis	BM	10.2	14.3	7.10	210	4	0.02%	<1	n.a.
#6	32	f	Suspected HαT **	BM	151	12.6	8.27	336	11	0.0001%	<1	n.a.
#7	30	f	ISM	BM	88.2	13.0	4.94	169	2	0.01%	<1	10
#8	62	m	SM-CMML	BM	70.7	9.0	13.91	122	4	0.008%	<1	5–10
#9	72	m	SM-CMML	BM	616	11.6	8.58	83	0	0.01%	<1	5
#10	69	f	Reactive Hypereosinophilia ***	PB	8.9	12.7	12.76	159	62	n.a.	n.a.	n.a.
#11	74	m	MPN/MDS-Eo	PB	5.8	9.4	34.27	24	36	n.a.	n.a.	n.a.
#12	78	f	Lymphocyte-Variant of Hypereosinophilic Syndrome	PB	6.8	10.9	9.91	373	26	n.a.	n.a.	n.a.

BM or PB were obtained at diagnosis or during follow-up. Percentage (%) of MC (for BM samples) or EO (for PB samples) were determined with FACS. Serum tryptase levels were routinely examined with fluoro-immuno-enzyme assay. * Although the patient did not fulfill all diagnostic criteria, the BM was hypoplastic and showed signs of aplastic anemia. ** In this patient, highly elevated tryptase levels were found but BM examinations did not reveal SM or other myeloid neoplasm. Unfortunately, the patient was lost for follow up. The most likely differential diagnosis is HαT, but a genetic test could not be performed. *** Accompanying a chronic kidney disease requiring hemodialysis. **^a^** Percentage of MC in MNC was analyzed using multicolor flow cytometry as described in the text. ^b^ Percentage of MC was assessed in Wright-Giemsa-stained BM smears. Abbreviations: BM, bone marrow; CMML, chronic myelomonocytic leukemia; EO, eosinophils; f, female; HαT, hereditary alpha tryptasemia; Hb, hemoglobin; ISM, indolent SM; m, male; MC, mast cells; MDS, myelodysplastic neoplasm; MNC, mononuclear cells; MPN, myeloproliferative neoplasm; n.a., not available; PB, peripheral blood; PLT, platelets; SM, systemic mastocytosis; and WBC, white blood cells. Normal ranges: basal serum tryptase, 0–11.4 ng/mL; Hb, 12–16 g/dL; WBC, 4.0–10.0 G/L; PLT, 150–350 G/L; and % EO in PB, 0–4%.

**Table 3:** Expression of coronavirus receptors on human mast cells (MC), basophils, and eosinophils.

Cell Type		Surface Expression of Coronavirus Receptors		
Cell Lines		CD13	CD26	CD147
HMC-1.1		+	−	++
HMC-1.2		+	−	++
MCPV-1.1		+	−	++
MCPV-1.2		+	−	++
MCPV-1.3		+	−	++
MCPV-1.4		+	−	++
ROSA^KIT WT^		+	−	+++
ROSA^KIT D816V^		+	−	+++
ROSA^KIT K509I^		+/−	−	+++
KU812		+	−	+++
EOL-1		+	+	+++
Primary cells	number of samples (n)			
Reactive BM MC	n = 6	+/−	+/−	+/++
Lung MC	n = 4	+	+/−	++
Skin MC	n = 3	+/++	+/−	++
Neoplastic BM MC	n = 3	+/−	+/−	++
Basophils (HD PB)	n = 13	+/++	+	+++
Eosinophils (HD PB)	n = 13	+	−	++
Eosinophils (HE PB)	n = 3	++	−	++

Expression of coronavirus receptors on various cell types (cell lines and primary cells) was examined using multicolor flow cytometry. Peripheral blood samples, bone marrow samples, or surgical tissue samples (lung, skin) were used as a source of primary cells. Results show the levels of expression of cell surface antigens (as staining index score defined below). The number (n) of tested samples (primary cells) in each group is also shown. Antibody reactivity was defined as staining index (SI): median fluorescence intensity (MFI) of tested antibody/MFI of corresponding isotype control. Scoring of SI: <1.3, −; 1.31–3, +/−; 3.01–10, +; 10.01–100, ++; >100, +++. +/++ in some donors the SI was 3.01-10 (+) and in others the SI was 10.01–100 (++). Abbreviations: BM, bone marrow; CD, cluster of differentiation; HD, healthy donor; HE, hypereosinophilia; MC, mast cells; and PB, peripheral blood.

**Table 4:** Expression of coronavirus receptor mRNA in cell lines as determined with qPCR.

Cell Line	Expression of mRNA Specific for					
	ACE2	ABL1	ABL2	ANPEP (CD13)	DPPIV (CD26)	BSG (CD147)
HMC-1.1	–	+	+	+	–	+
HMC-1.2	–	+	+	+	–	+
ROSA^KIT WT^	–	+	+	+	–	+
ROSA^KIT D816V^	–	+	+	–/+	–	+
ROSA^KIT K509I^	–	+	+	–	–	+
MCPV-1.1	–	+	+	+	–	+
MCPV-1.2	–	+	+	+	–	+
MCPV-1.3	–	+	+	+	–	+
MCPV-1.4	–	+	+	+	–	+
KU812	–	+	+	+	–	+
EOL-1	–	+	+	–	+	+

mRNA expression of various cell lines was evaluated using qPCR, and *GUSB* served as a reference gene; +, mRNA expression >3% of GUSB mRNA; −/+, mRNA expression 1.49–3% of GUSB mRNA; −, mRNA expression <1.49% of GUSB mRNA. Abbreviations: ACE, angiotensin-converting enzyme; ABL, Abelson tyrosine kinase; ANPEP, aminopeptidase N; BSG, basigin; CD, cluster of differentiation; DPPIV, dipeptidylpeptidase IV; and qPCR, quantitative real-time PCR.

**Table 5:** Effects of drugs on surface expression of coronavirus receptors (CoV-R).

Cell Line	CoV-R	Effects of Drugs on Expression of CoV-R		
		Hydroxychloroquine	Dexamethasone	Vitamin D
HMC-1.1	CD13	69 ± 5.0	53 ± 14.7 *	88 ± 2.8
	CD147	100 ± 6.6	63 ± 19.2 *	103 ± 5.7
HMC-1.2	CD13	104 ± 11.7	94 ± 3.9	80 ± 19.0
	CD147	100 ± 2.4	99 ± 2.4	102 ± 7.7
ROSA^KIT WT^	CD13	104 ± 2.5	95 ± 5.9	106 ± 7.9
	CD147	101 ± 11.4	87 ± 10.5	107 ± 4.8
ROSA^KIT D816V^	CD13	117 ± 15.6	110 ± 15.4	90 ± 19.8
	CD147	94 ± 14.5	90 ± 12.3	83 ± 35.5
ROSA^KIT K509I^	CD13	100 ± 8.3	98 ± 5.2	89 ± 13.2
	CD147	121 ± 27.1	108 ± 27.0	89 ± 41.8
MCPV-1.1	CD13	98 ± 2.2	84 ± 4.2	101 ± 6.3
	CD147	91 ± 5.5	87 ± 9.4	101 ± 5.2
MCPV-1.2	CD13	113 ± 9.4	91 ± 6.2	115 ± 26.4
	CD147	99 ± 4.5	106 ± 21.5	118 ± 14.0
MCPV-1.3	CD13	100 ± 6.4	86 ± 7.3	113 ± 7.3
	CD147	83 ± 13.3	90 ± 8.2	96 ± 22.5
MCPV-1.4	CD13	107 ± 3.7	89 ± 3.0	108 ± 12.0
	CD147	95 ± 8.4	90 ± 5.1	111 ± 11.3
KU812	CD13	98 ± 5.8	98 ± 1.0	98 ± 4.2
	CD147	103 ± 3.6	105 ± 9.2	106 ± 10.9
EOL-1	CD13	110 ± 8.5	92 ± 5.3	109 ± 12.1
	CD26	67 ± 18.0 *	90 ± 5.0	81 ± 13.0
	CD147	103 ± 5.2	106 ± 4.0	107 ± 0.9

The mast cell lines HMC-1, ROSA, and MCPV-1, the basophilic cell line KU812, and the eosinophilic cell line EOL-1 were incubated in a control medium or a medium supplemented with 10 µM hydroxychloroquine, 1 µM dexamethasone, or 5 µM vitamin D for 24 h at 37 °C. Cells were stained with PE-conjugated antibodies against the CoV-R, CD13 (aminopeptidase N), CD26 (dipeptidyl-peptidase IV), and CD147 (basigin). Expression of CoV-R was determined using multicolor flow cytometry as median fluorescence intensity (MFI). Results represent the mean ± S.D. percent of untreated control of 3–6 independent experiments. Asterisk (*): *p* < 0.05 compared to control as assessed by one-way ANOVA followed by Bonferroni’s post hoc comparison test. Abbreviations: ANOVA, analysis of variance; CD, cluster of differentiation; CoV-R, coronavirus receptor; and PE, phycoerythrin.

## Data Availability

The data used and/or analyzed during the current study are available from the corresponding author on reasonable request.

## Supplementary Materials

The following supporting information can be downloaded at: https://www.mdpi.com/article/10.3390/cells13020173/s1, Figure S1: Effects of hydroxychloroquine on the expression of CD26 on EOL-1 cells; Table S1: Specification of monoclonal antibodies used in this study; Table S2: Primer sequences used for quantitative real-time PCR.
