# Supplementary material for: Coronavirus Receptor Expression Profiles in Human Mast Cells, Basophils, and Eosinophils
Source: Cells. 2024 Jan 17;13(2):173. doi: 10.3390/cells13020173 (PMC10814915; doi:10.3390/cells13020173)
Supplement: Supplementary file 1 [file cells-13-00173-s001.zip › cells-2788293-supplementary.pdf]

Supplementary Data to Manuscript:

Coronavirus Receptor Expression Profiles in Human Mast Cells, Basophils and Eosinophils (Degenfeld-Schonburg et al.)

Supplemental Material and Methods

Quantitative polymerase chain reaction (qPCR)

RNA was isolated from mast cell (MC)-related cell lines (HMC-1.1, HMC-1.2, ROSA$^{KIT\ WT}$, ROSA$^{KIT\ D816V}$, ROSA$^{KIT\ K509I}$, MCPV-1.1 through MCPV-1.4), the basophil (BA) cell line KU812, and the eosinophil (EO)-related cell line EOL-1, using the RNeasy MinElute Cleanup Kit or RNeasy Plus Mini Kit (Qiagen, Hilden, Germany). cDNA was synthesized using Moloney murine leukemia virus reverse transcriptase and random primers (both from Invitrogen, Carlsbad, CA). To confirm expression of transcripts (mRNA) specific for aminopeptidase-N (CD13), dipeptidylpeptidase IV (DPPIV, CD26), basigin (BSG, CD147), ACE2, ABL1, and ABL2 in our cell lines, qPCR experiments were conducted as reported [1-3] using primers listed in Supplementary Table S2. qPCR was performed on a QuantStudio 3 Real-Time PCR System (Thermo Fisher Scientific, Waltham, MA, USA) using iTaq Universal SYBR Green Supermix (Bio-Rad, Hercules, CA, USA) and plasmid standards. CD13, CD26, CD147, ACE2, ABL1, and ABL2 mRNA copy numbers were normalized to beta-glucuronidase (GUSB) mRNA copy numbers and were expressed as percent of GUSB. The results are shown as a semi-quantitative scoring system to discriminate between clearly positive expression (+, >3%), clearly negative results (-, <1.49%) and slightly positive expression (+/-, 1.49%-3%).

Supplementary Figures

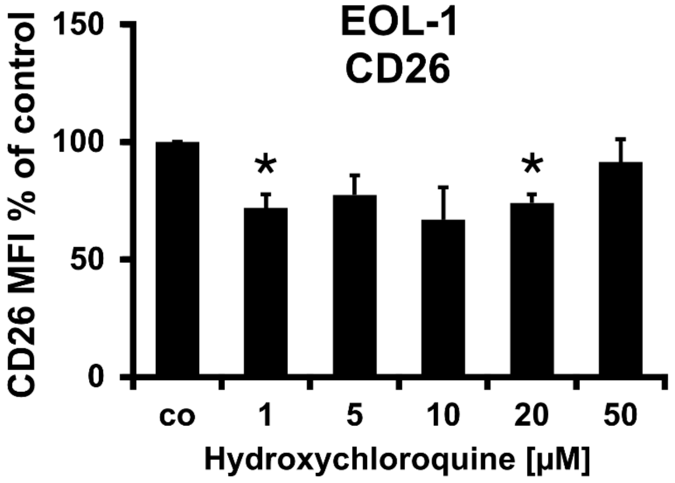

Supplementary Figure S1.

Effects of hydroxychloroquine on the expression of CD26 on EOL-1 cells.

EOL-1 cells were incubated in control medium alone (co) or medium supplemented with different concentrations of hydroxychloroquine (1–50 µM) for 24 hours at 37°C. Thereafter, cells were stained with the PE–conjugated antibody M-A261 directed against the CoV–R, CD26. Expression of CD26 was determined by flow cytometry measuring the median fluorescence intensity (MFI). CD26 expression levels are expressed as MFI in percent of control. Results represent the mean ± S.D. of 3 independent experiments. Asterisk (*): $P<0.05$ compared to control as assessed by one-way ANOVA followed by Bonferroni´s post hoc comparison test. The solvent control (DMSO) did not show any downregulating effect on expression of CD26 on EOL-1 cells (112±13% of medium co). Abbreviations: CoV-R, coronavirus receptor; ANOVA, analysis of variance; DMSO, dimethyl sulfoxide.

Supplementary Tables

Supplementary Table S1

Specification of Monoclonal Antibodies used in this Study

CD	Antigen	Clone	Isotype	Conjugate	Specificity	Company
n.c.	isotype control	MOPC-21	mouse, IgG1	PE	human	BD Biosciences
n.c.	isotype control	20102	mouse, IgG2A	PE	human	R&D Systems
CD13	APN	WM15	mouse, IgG1	PE	human	BD Biosciences
CD14	LPSR	TÜK4	mouse, IgG2A	FITC	human	Dako
CD26	DPPIV	M-A261	mouse, IgG1	PE	human	BD Biosciences
CD34	HPCA-1	581	mouse, IgG1	PB	human	BioLegend
CD38	T10	HIT2	mouse, IgG1	APC	human	BD Biosciences
CD45	LCA	HI30	mouse, IgG1	APC-Cy7	human	BioLegend
CD45	LCA	2D1	mouse, IgG1	PerCP	human	BD Biosciences
CD45	LCA	HI30	mouse, IgG1	V500	human	BD Biosciences
CD117	SCFR/KIT	104D2	mouse, IgG1	PE-Cy7	human	invitrogen
CD123	IL-3RA	6H6	mouse, IgG1	PE-Cy7	human	BioLegend
CD147	BSG	HIM-6	mouse, IgG1	PE	human	BioLegend
CD147	BSG	HIM-6	mouse, IgG1	PE	human	BD Biosciences
CD203c	E-NPP3	FR3-16A11	mouse, IgG1	APC	human	Miltenyi Biotec
n.c.	Siglec-8	837535	mouse, IgG1	PE	human	R&D Systems

Abbreviations: APN, aminopeptidase N; APC, allophycocyanin; BD, Becton Dickinson; BSG, basigin; CD, cluster of differentiation; Cy, cyanine dye; DPPIV, dipeptidyl-peptidase IV; E-NPP3, ectonucleotide pyrophosphatase/phosphodiesterase-3; FITC, fluorescein isothiocyanate; HPCA-1, human precursor cell antigen-1; Ig, immunoglobulin; IL-3RA, interleukin-3 receptor alpha chain; LCA, leukocyte common antigen; LPSR, lipopolysaccharide; n.c., not (yet) clustered; PB, pacific blue; PE, phycoerythrin; PerCP, peridinin chlorophyll protein; SCFR, stem cell factor receptor.
Company Locations: BD Biosciences, San José, CA, USA; BioLegend, San Diego, CA, USA; Dako, Glostrup, Denmark; Miltenyi Biotec, Bergisch Gladbach, Germany; R&D Systems, Minneapolis, MN, USA.

Supplementary Table S2

Primer sequences used for quantitative real time PCR

Gene		Sequence (5´-> 3´)
ACE2	forward	CAAAGTTCACTTGCTTCTTGGA
	reverse	ACCATTTGTCCCCAGCATTA
ABL1	forward	TGTATGATTTTGTGGCCAGTGGAG
	reverse	GCCTAAGACCCGGAGCTTTTCA
ABL2	forward	TGGACAGCACCAGAGAGTCTT
	reverse	GGTAGCAATTTCCCACAACAA
ANPEP (CD13)	forward	GCGAGTACATGGAGGGCAAT
	reverse	TCGAAGCATGGGAAGGACTT
DPPIV (CD26)	forward	TCAATATCTCCTGATGGGCAGTTT
	reverse	TGTCATATGAAGCTGTGTAGGAATGC
BSG (CD147)	forward	TCACTGACTGGGCCTGGTA
	reverse	TCACGAAGAACCTGCTCTCG
GUSB	forward	GGGCTTCGAGGAGCAGTGG
	reverse	GCTGGAGGGAACTGGCATGT

Abbreviations: PCR, polymerase chain reaction.

Supplementary References

1. Smiljkovic, D.; Herrmann, H.; Sadovnik, I.; Gamperl, S.; Berger, D.; Stefanzl, G.; Eisenwort, G.; Hoermann, G.; Kopanja, S.; Dorofeeva, Y.; et al. Expression and regulation of Siglec-6 (CD327) on human mast cells and basophils. J. Allergy Clin. Immunol. 2023, 151(1), 202-211, doi:10.1016/j.jaci.2022.07.018.
2. Herrmann, H.; Cerny-Reiterer, S.; Gleixner, K.V.; Blatt, K.; Herndlhofer, S.; Rabitsch, W.; Jäger, E.; Mitterbauer-Hohendanner, G.; Streubel, B.; Selzer, E.; et al. CD34(+)/CD38(-) stem cells in chronic myeloid leukemia express Siglec-3 (CD33) and are responsive to the CD33-targeting drug gemtuzumab/ozogamicin. Haematologica 2012, 97, 219-226, doi:10.3324/haematol.2010.035006.
3. Herrmann, H.; Sadovnik, I.; Cerny-Reiterer, S.; Rülicke, T.; Stefanzl, G.; Willmann, M.; Hoermann, G.; Bilban, M.; Blatt, K.; Herndlhofer, S.; et al. Dipeptidylpeptidase IV (CD26) defines leukemic stem cells (LSC) in chronic myeloid leukemia. Blood 2014, 123, 3951-3962, doi:10.1182/blood-2013-10-536078.
